# Edible Flowers Used in Some Countries of the Mediterranean Basin: An Ethnobotanical Overview

**DOI:** 10.3390/plants11233272

**Published:** 2022-11-28

**Authors:** Riccardo Motti, Bruno Paura, Alessia Cozzolino, Bruna de Falco

**Affiliations:** 1 Department of Agricultural Sciences, University of Naples Federico II, Via Università, 100 Portici, 80055 Naples, Italy; 2 Department of Agricultural, Environmental and Food Sciences, University of Molise, 86100 Campobasso, Italy; 3 Spanish Bank of Algae, Marine Biotechnology Center, University of Las Palmas de Gran Canaria, Muelle de Taliarte s/n, 35214 Telde, Spain

**Keywords:** food plants, new foods, food system, nutrition, diet, bioactive compounds, aroma, antioxidant activity

## Abstract

Edible flowers are becoming an essential component of people’s nutrition in the Mediterranean basin. In the last decades, many researchers also have focused their attention on the nutritional composition of the edible flowers, as well as their antioxidant and antimicrobial properties, including studies on their safety issues. Despite the growing interest in the use of flowers in human nutrition, the ethnobotanical literature is lacking coverage of some important issues, particularly those which concern the use of flowers in the folk tradition. Only recently, a review regarding the contribution of 32 edible flowers to the Mediterranean diet was published. The aim of the present review is to document the plant lore regarding the wild and cultivated edible flowers consumed in the Mediterranean basin. Based on the 112 studies reviewed, we documented 251 taxa as being used in the Mediterranean basin as edible flowers. The plant species belong to 45 families and 141 genera. Asteraceae (54 taxa) is the most frequently cited family. *Sambucus nigra* L. is the most cited species. This study can be the basis for future research on the supposed bioactivity and toxicity of wild and cultivated flowers.

## 1. Introduction

Humans have gathered wild edible plants since ancient times, and the plants have become part of the human diet and traditional food systems [1]. Eating flowers is a legacy of the many cultures that have been using wild edible plants in their food traditions for centuries [2]. Many species of edible flowers were already used in ancient Greece and Rome, in medieval France, and Victorian England as relishes and flavor enhancers of many dishes [3,4]. Many ancient texts refer to edible flowers. For example, the Bible cites dandelions as one of the “bitter herbs” eaten as salads, while in the Song of Solomon saffron, the stamens of *Crocus sativus*, is mentioned [5]. In Italy, evidence on the use of flowers can be found in some refined preparations, such as, for example, *vino violatum* (violet wine) or *rosatum* (rose petal wine), safflower flower sauce, and marjoram flower meatballs, whose recipes are reported in *Apicius De Re Coquinaria*, a famous cookbook from imperial Rome (1st century AD) [6]. During the Middle Ages, the marigold flower was a common ingredient in salads, and its flowers appeared in numerous preparations. In fact, during the Renaissance (15th and 16th centuries) cooking with flowers and making candied fruits was a very common practice. In his *Libro de arte Coquinaria*, the cook Maestro Martino reported in his recipe book a “menestra de fior de sambuco” (elderflower soup), [7] while Bartolomeo Scappi presented the preparation for a rose water and a borage flower confetto [8]. From the Baroque period, we can mention Gerolamo Mei, who reported numerous recipes based on flowers, including biscuits with *Citrus aurantium* flowers, violet syrups, and pink sugar, composed of violets, hyssop, and roses [9]. Flowers have traditionally been used in cooking in various cultures, not only for their aesthetic appearance but also for their specific taste and smell [10]. Edible flowers are traditionally consumed in salads or used to prepare cakes, fritters, drinks, teas, and liqueurs, served as aroma enhancers, or as a side dish. In many cultures, fresh flowers are eaten as snacks, especially by children, for the sweet taste due to the nectar. Nowadays, the interest in the use of edible flowers is increasing, especially among chefs, not only for their aesthetic properties but also because of their proven health benefits [11,12]. The search for new food products is also a pursuit of new colors, textures, and flavors that can be achieved with the use of edible flowers [13]. Moreover, consumers are increasingly choosing food products containing natural ingredients and edible flowers to bring interesting elements to culinary and dietary habits [14]. From this perspective, the interest in edible flowers is continuously increasing, and many researchers have focused their attention on the nutritional composition, including the acceptability, the antioxidant and antimicrobial activities, the effects on human health, and the safety issues [15]. However, because of the low availability (i.e., short blooming period and in limited places) and poor post-harvest life, edible flowers are commonly utilized by the local people during their respective flowering period only. The use of flowers as food cannot, however, be considered a new discovery, but a rediscovery of ancient ethnobotanical traditions [16]. The role of ethnobotanical research is, in fact, to avoid the loss of the traditional knowledge concerning plant lore, and in this context, the ethnobotany of food plants is a fairly well-developed research field in several European geographical areas and social communities [17,18,19,20]. In this scenario, according to Pieroni et al. [21], focusing on the traditional uses of edible flowers can constitute an important tool for analyzing and preserving the traditional ecological knowledge (TEK) and cultural diversity in the Mediterranean basin. Despite the growing interest in the use of flowers in human nutrition, the ethnobotanical literature is lacking coverage of some important issues, particularly those which concern the use of flowers in the folk tradition. Only recently, a review regarding the contribution of edible flowers to the Mediterranean diet was published [22]. In this review, the phytonutrients, the bioactivity evaluation, and the applications of only 32 species were taken into consideration. In this context, we review the available ethnobotanical literature in order to obtain as many data as possible on the use of edible flowers by the populations living in the countries of the Mediterranean basin. Specifically, the main objectives of the present study were to:

- Document the folk knowledge regarding the wild and cultivated edible flowers used in the Mediterranean basin;
- Underline the uses of the most cited edible flowers in the Mediterranean folk tradition;
- Highlight the chemical composition and traditional therapeutical uses of the most reported edible flowers.

## 2. Results

Based on the literature review, 251 taxa are documented as being used as food plants by traditional users in the countries bordering the Mediterranean basin (Table 1).

**Figure 1:**
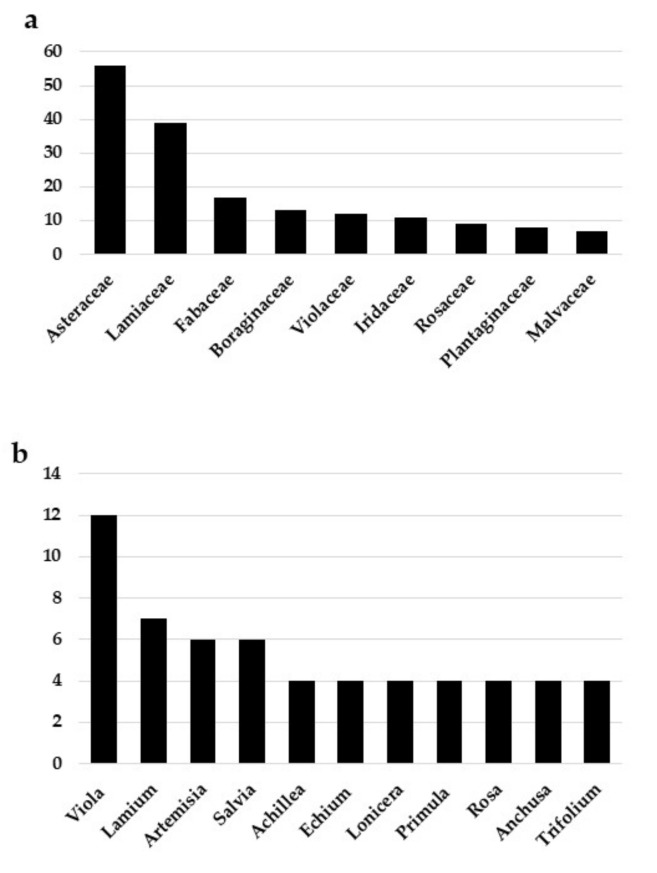
Most cited families and number of species per family (**a**); most cited genera and number of species per genus (**b**).

The cited taxa belong to 45 families and 141 genera. Asteraceae (54 taxa) is the most frequently cited family, followed by Lamiaceae (39) and Fabaceae (17) (Figure 1a). The genus Viola is the most abundant in species (12), followed by Lamium (7) and Artemisia and Salvia (6) (Figure 1b).

**Figure 2:**
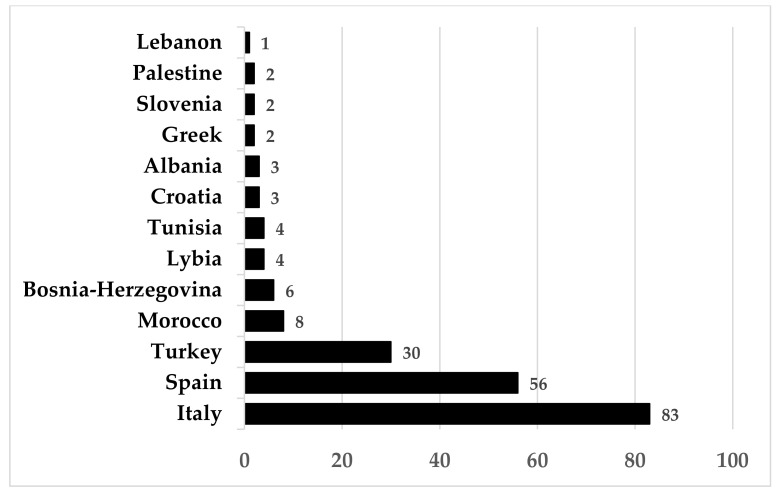
Number of edible flower species used for each Mediterranean country.

As shown in Figure 2, from the analyses carried out at a national scale, Italy has the largest number of species used in a single country (83), followed by Spain (56) and Turkey (30).

The elderberry (*Sambucus nigra* L.) is the most cited species (20 papers, five countries); it is a deciduous shrub native to Europe, introduced into various parts of the world, including E. Asia, N. America, New Zealand, and the southern part of Australia [109]. By the action of birds, its seeds are rapidly spread, colonizing the forest edges, areas along the roads, rails, and fence lines. Elderberry shrubs bloom over the summer from June to August, depending on the climate. The white, scented flowers are grouped in large corymbs. The elderberry flowers (such as the related *S. racemosa* L.) are dipped into a light batter and then fried to make fritters or used in the preparation of pancakes and omelettes. The flowers are also used for the preparation of juice, jam, jellies, and beverages and as an aromatizer. The odor of elderflowers has been shown to be related to the occurrence of 59 compounds: cis-Rose oxide, nerol oxide, hotrienol, and nonanal contribute to the characteristic elderflower odor, whereas linalool, α-terpineol, 4-methyl-3-penten-2-one, and (Z)-β-ocimene contribute with floral notes [110]. The flower extract has a higher content of phenolic compounds, such as rutin, chlorogenic acid, and rosmarinic acid [111]. Although currently they are mainly used in the food industry as flavoring agents due to their phytochemical composition and related bioactivities, elderflowers or their extracts are becoming attractive for other uses, such as food supplements and nutraceutical ingredients, and as raw materials for the pharmaceutical industries. The beneficial health-promoting effects of elderflowers are well known, including effects against degenerative diseases (cardiovascular and inflammatory diseases), cancer, and diabetes, and also present antioxidant, anti-inflammatory, immune-stimulating, chemo-preventive, and atheroprotective effects [112]. *S. nigra* flowers are widely used also in the folk phytotherapy in Albania, Algeria, Italy, and Spain: the internal use is applied to the treatment of bronchial diseases, colds, and abdominal pains and as an anti-inflammatory, or they are used as antipyretics, diuretics, digestives, diaphoretics, anti-rheumatics, and galactagogues (e.g., [113,114,115,116,117]). As a topical application, the flowers are used for the treatment of conjunctivitis, wounds, burns, and rheumatic pains (e.g., [32,118,119]). The elderberry flower infusion is also used as a skin toner and whitener [120].

The black locust or false acacia (*Robinia pseudacacia* L.) is a deciduous tree native to North America and naturalized elsewhere in temperate areas of Europe, Southern Africa, and Asia and is considered an invasive species in some areas. This species was probably introduced to Europe in 1601 [121]. In several European databases, it is classified as highly invasive and is now listed amongst the 40 most invasive woody angiosperms globally [122]. In the time of flowering (May and June), black locust flowers are one of the most important sources of honey production [123]. *R. pseudacacia* flowers (14 papers, five countries) are commonly used in the preparation of omelettes, fritters, syrup, and liqueur and are also eaten as snacks. The chemical composition shows 24.55% protein, 8.51% ash, 40.97% total sugar, and 160.44 mg of ascorbic acid on a dry matter basis, respectively. The free sugar is mainly composed of fructose, sucrose, and glucose [124]. Linalool, cis-β-ocimene, methyl anthranilate, phenyl ethyl alcohol, germacrene D, (E)-α-bergamotene, benzeneacetic acid methyl ester, (Z)-nerolidol, and indole are important contributors to the pleasant aroma of the flowers of *R. pseudacacia* [125]. The black locust flower polyphenolic extract contains a significant percentage of polyphenolic compounds and presents good antioxidant and antitumoral activity [126]. *R. pseudacacia* flowers are used in Italy as an infusion for the treatment of flu or as sedative [24,39], while in Turkey the infusion is used as a generic product which is good for health [127].

The borage or starflower (*Borago officinalis* L.) is an annual herb widely distributed beyond its original habitat in the Euro-Mediterranean region as a wild weed or cultivated as a garden plant, a crop vegetable, or for medicinal purposes [128,129]. The flowers are blue and rarely appear white or rose colored. The flowers arise along scorpioid cymes to form large floral displays. The flowering period is from early spring to summer (in some Italian regions even in winter), depending on the climate [130]. Borage flowers (14 papers, three countries) are used in salads, fritters, and soups or as a vinegar aromatizer. Aldehydes and terpenes are the major chemical classes among the aromatic volatile components of the B. officinalis flowers [131]. Borage flowers are rich in fatty acids (mainly α-linolenic, stearidonic, palmitic, linoleic, and γ-linolenic acids), organic acids (mainly malic and levulinic acids), and carotenoids (β-carotene and lutein) [132]. Borage flowers could therefore be considered as a source of putative antioxidant and antibacterial compounds to improve human health and to be used as a biopreservative in food and cosmetic industries [133]. The infusion or macerate of borage flowers is used in Italy and Spain for the treatment of colds, bronchitis, sore throats, and gastritis; it is also used as a diuretic and an anti-rheumatic [48,134].

The caper bush or flinders rose (*Capparis orientalis* Veill; *C. spinosa* L.) is a deciduous shrub, apparently native to the dry regions of western and central Asia; however, long ago it spread to North and East Africa, Madagascar, Australia, and Oceania [135]. The branched stems are trailing or ascending. These plants prefer dry heat and intense sunlight. The drought- and salt-tolerant nature of these species allows it to persist in a wide range of habitats, even on nutrient-poor, sandy, and gravelly soils [136]. The flowers are sweetly fragrant, white and often tinged with pink, with many long violet-colored stamens. The caper bush has been introduced as a specialized culture in some European countries. The flower buds (12 papers, five countries) are consumed salted or pickled as a vegetable condiment and are among the most popular species of aromatic plants grown in the Mediterranean zone. The flower buds are rich in volatile compounds, and cinnamaldehyde and benzaldehyde are the most abundant aldehydes [137]. Methyl isothiocyanate and dL-limonene are the main aroma-active compounds of the fresh flower buds [138]. The amounts of the flavonoids kaempferol and quercetin 3-O-glucoside, quercetin 3-O-glucoside-7-O-rhamnoside, and rutin in the caper’s buds are remarkable [139]. A caper decoction is used in Tunisia as antidiabetic and diuretic [63], while in Turkey it is used as a treatment for hemorrhoids and gastric ulcers [23].

The dandelion (*Taraxacum officinale* Weber and F.H. Wigg.) is a herbaceous perennial plant native to Europe and Asia that can thrive in a wide range of conditions; in fact, it can be found on all the continents, except for Antarctica [140]. The leaves are arranged in a basal rosette, yellow to orange flowers are grouped in solitary capitula at the top of the scape. Blooming occurs from spring until autumn, depending on the plant’s location. Dandelion flowers (eight papers, four countries) are used in popular traditions for the preparation of salads, fritters, risotto, jam, and tea or as a seasoning. Dandelion flowers are rich in phytochemicals, such as carotenoids, flavonoids, phenolic acids, and terpenes, with the resulting sesquiterpene lactones and caffeoylquinic acid derivatives being the most abundant secondary metabolites, followed by flavonoids [141]. The antioxidant and cytotoxic properties can in part be attributed to the presence of luteolin and luteolin 7-glucoside [142,143]. Dandelion flowers (*T. officinale*, as well as the related *T. campylodes* G.E. Haglund) are used in the folk phytotherapy of the Mediterranean basin in infusion for the treatment of respiratory or urogenital diseases [39,44] or topically for healing wounds [28,104].

White and red clovers (*Trifolium repens* L. and *T. pratense* L., respectively) are a globally distributed species of perennial herbs which are common in most grassy areas or are cultivated as a forage crop. The flowers are whitish (*T. repens*) or dark pink with a paler base (*T. pratense*); they are produced in a dense inflorescence and are mostly visited by bumblebees [144]. Blooming occurs from early spring until late summer, depending on the climate. Clovers flowers (11 papers, three countries) are used in salads, cakes, fritters, and soups or for tea preparation. *Trifolium* extracts have a high total content of polyphenols as well as a high antioxidant potential [145]. *T. repens* extract contains a high level of rutin and quercetin, while *T. pratense* extract contains luteolin and kaempferol; these data support the use of clover flowers as healthy food ingredients [146]. *T. pratense* flowers are used in Italy and Turkey in the treatment of stomach diseases, coughs, and menopause disorders [29,106,147].

The sweet or English pansy (*Viola odorata* L.) is a herbaceous perennial plant native to the south and parts of western Europe and is now widely naturalized. *V. odorata* is a rosette-forming plant with long, freely-rooting stolones. The flowers are dark violet or white and sweet-scented and appear in spring. Sweet pansies have been cultivated for cosmetics and medicine in Europe since antiquity [148]. The sweet pansy flowers (nine papers, four countries) are consumed in salads or are used to prepare sweets, fritters, and liqueurs. Flavonol glycosides, principally derivatives of kaempferol, are among the major chemical constituents of the sweet pansy, and the presence of high amounts of free sugars and mucillage is reported [149]. The sweet pansy flower infusion is commonly used in Italy against coughs and as a diaphoretic, a diuretic, or a mild laxative or as cold adjuvant [150,151].

The wild pansy or heartsease (*Viola tricolor* L.) is a biennial or a short-lived perennial which is native to Europe and Asia. The flowers can be purple, blue, yellow, or white and appear from spring to late summer. Wild pansy flowers are usually added to salads in Italy and Bosnia-Herzegovina (four papers, three countries). *V. tricolor* flowers show high contents of anthocyanidins and flavonoids; the highest cyanidin-3-glucoside content is present in the violet flower, while the white and yellow pansies showed the highest rutin content [152]. The *V. tricolor* flower infusion is taken orally in the Italian traditional pharmacopoeia for the treatment of coughs [29].

The corn poppy (*Papaver rhoeas* L.) is a cosmopolitan annual herbaceous plant. Before anthesis, the elliptical flower buds are pendulous, but when it occurs, they become erect and the two sepals underneath drop, allowing the red petals to expand. This species has been associated with agriculture in the Old World since early times, and its diffusion is linked to the cultivation of cereals [130]. Corn poppy flowers (five papers, three countries) are used raw or cooked in the preparation of sorbet, patties, or as a stew or egg–vegetable dish. Various phytochemical components have been identified in corn poppy petals (e.g., alkaloids, flavonoids, vitamins, anthocyanins, and essential oils); the petals are rich in anthocyanins, which are responsible for the red color [153]. The most represented anthocyanins in the extracts of P. rhoeas were found to be delphinidin-3-O-glucoside, cyanidin-3-O-glucoside, cyanidin-3-O-rutinoside, peonidin-3-O-glucoside, petunidin-3-O-glucoside, petunidin-3- acetylglucoside, and delphinidin-3-p-coumaroylglucoside [154]. Different parts of the plant (the roots, stems, leaves, and petals) exhibited several biological activities, including antidepressant, antimicrobial, antioxidant, antiulcerogenic, and cytotoxic activities [155]. The *P. rhoeas* flowers are widely used in the Mediterranean basin as a sedative and for the treatment of various ailments, such as respiratory and gastro-intestinal system ailments, diabetes, and measles, and topically as a vulnerary [156,157,158].

The pot marigold (*Calendula officinalis* L.) is an annual or short-lived perennial herb whose origin is unknown, but it is probably native to southern Europe and the eastern Mediterranean area. It is a weed that grows in cultivated fields, along roadsides, and in disturbed sites on a variety of soil types [159]. The marigold is widely cultivated as an ornamental and for its therapeutic properties. The daisy-like inflorescences are typically bright orange or yellow and held on thick stems. The marigold blooms over a long period where the conditions are suitable. The *C. officinalis* flowers (as well as the related *C. arvensis* L.) are widely used in Italy and Bosnia-Herzegovina in salads or as a condiment. The main constituents of the marigold flowers include steroids, terpenoids, triterpenoids, flavonoids, phenolic acids, and carotenes [160,161]. Faradiol, caffeic acid, rutin, and chlorogenic acid isolated from C. officinalis exhibit biological activity [162]. Pharmacological studies have shown that the marigold exhibits antibacterial, antiviral, anti-inflammatory, antioxidant, hypoglycemic, hypolipidemic, and wound healing properties [163,164,165]. In the folk phytotherapy, both *C. officinalis* and *C. arvensis* are used as remedies for a wide range of diseases. The *C. arvensis* flower infusion is used orally in Spain as an emmenagogue [137], in Greece as an antispasmodic [166], and in Italy as an antispasmodic and a diuretic [167]. The *C. officinalis* flower infusion is used orally in Italy for urinary tract disorders, gastrointestinal pains, and dysmenorrhea [28,168] and in Croatia for kidney disorders, hepatitis, and stomach ulcers [169]. Both marigolds are used topically in the Mediterranean basin for skin disease treatment (wounds, burns, erythema, rheumatic pains, varicose veins, corns, warts, etc.) (e.g., [28,97]).

Finally, the use of large-leaved linden (*T. platyphyllos* Scop.) flowers for tea and liqueur preparation is worthy of note (five papers, four countries). This species is native to central and southern Europe and is widely planted throughout the temperate world as an ornamental tree [170]. The very fragrant, yellowish-white flowers are arranged in drooping, cymose clusters and appear in late spring to early summer. A detailed phytochemical profile of *T. platyphyllos* inflorescences revealed the presence of flavonoids, mainly quercetin glycosides (rutin, hyperosid, quercitrin, quercetin-3,7-di-O-rhamnoside, quercetin-rhamno-xyloside, and quercetin-3-O-gluco-7-O-rhamnoside) and kaempferol glycosides (astragalin, tilirosid, kaempferol- 3-O-gluco-7-O-rhamnoside, and kaempferol-3,7-di-O-rhamnoside) [171]. A high content of oligomeric and polymeric procyanidins, mainly composed of catechin and epicatechin building blocks such as prodelphinidin C and procyanidin B4, has been identified [172]. The *T. platyphyllos* flower infusion is widely used in the folk phytotherapy of the Mediterranean basin for its sedative properties but also to treat coughs, sore throats, and bronchitis and as a febrifuge or galactagogue [173,174,175].

## 3. Discussion

The studies we included in this review demonstrate that the edible flowers are widely used for human nutrition throughout the Mediterranean basin countries. Their use is closely linked to both the local floras and the traditional knowledge. In fact, with the exception of *Capparis orientalis*, *Robinia pseudacacia*, *Taraxacum officinale*, *Tilia platyphyllos*, and *Viola odorata*, no species is reported for more than two or three countries, and 79% of the species are mentioned only once. The flowers of some species which are cultivated to be ornamental and are sometimes naturalized (*Opuntia ficus-indica*, *Tilia tomentosa*, *Vachellia farnesiana*, *Rosa* × *centifolia*, and *Rosa foetida*) are also used as food.

Edible flowers are also identified as functional foods for their nutraceutical properties and, in particular, for their content of antioxidant compounds, which can play an important role in promoting health and preventing different diseases. Many studies (e.g., [176,177]) have highlighted that a dietary antioxidant intake has a protective effect against free radical-related pathologies, such as cardiovascular diseases, cancer, and chronic respiratory and neurodegenerative diseases. Recent studies have also highlighted that the protective effect of nutraceuticals is linked to the association of several phytochemical molecules at low concentrations, as they occur naturally in the diet [178]. In addition, edible flowers are promising raw materials for the prevention or improvement of skin aging, immunosenescence, and neurodegeneration, thanks to active ingredients such as flavonoids, phenolic acids, carotenoids, phenylethanoid glycosides, polysaccharides, etc. [179].

As for the other plant parts, a correct identification and a deep knowledge of the species is of fundamental importance for the consumption of the flowers as food because some of them are potentially toxic or poisonous. In this regard, the use report of the flowers of *Ferula communis* in Palestine as a vegetable [89] is noteworthy. As highlighted by Akaberi et al. [180], this species is characterized by different chemical constituents; the toxic chemotype mainly produces prenylated coumarins such as ferulenol that are responsible for a lethal hemorrhagic disorder called ferulosis, while the non-toxic chemotype contains daucane-type sesquiterpenoids such as ferutinin. *Echium vulgare* flowers are reportedly eaten as a snack in Spain [20]. Lucchetti et al. [181] highlighted that the nectar of this species contains pyrrolizidine alkaloids, toxic compounds that can be a potential human health risk. As highlighted by Amrouche et al. [22], to date most of the edible flowers consumed in the Mediterranean diet are non-toxic at low doses, but high doses might cause toxicity in multiple organs. In this regard, new investigations are aimed at specific knowledge of wildflower phytochemistry and at recommending quantities for consumption that are desirable. On the other hand, the issues of potentially toxic elements (i.e., pollution, pesticides) or dangerous microorganisms should be taken into account in the consumption of edible flowers. Hazardous bacteria may come from both the agricultural production and the food chain, while the sources of chemical impurities are mostly agricultural production and the environment [14] Therefore, it is always advisable to ascertain the origin of the products to be consumed and also to consider that they are often used raw and that washing can damage them.

Edible flowers can also play an important role in traditional gastronomy because they can be used in the recipes of many local dishes, and in a certain way, they can contribute to the cultural identity of some geographical areas. According to Jordana [182], in order to be traditional, a product must be linked to a territory, and it must also be part of a set of traditions which will necessarily ensure its continuity over time. The potential of edible flowers should be further explored for the possible economic opportunities that could be generated for local gatherers and communities. The diversification of production using such resources could be a socio-economically sustainable activity in areas with non-optimal farming conditions by contributing to population stabilization in rural areas.

## 4. Materials and Methods

**Figure 3:**
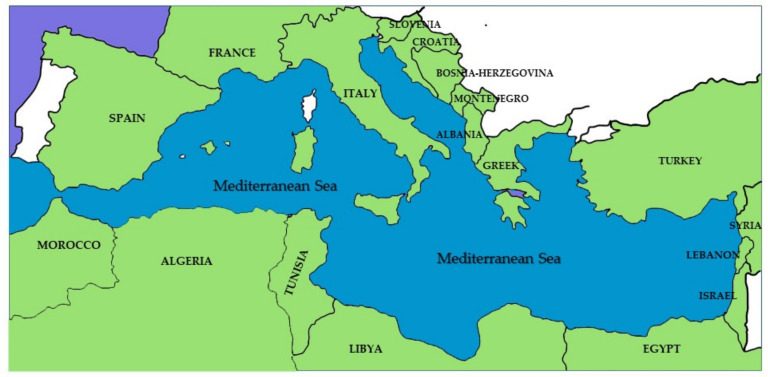
Mediterranean basin and its bordering countries.

A comprehensive ethnobotanical literature search on the food plants used in the Mediterranean area was carried out using existing online scientific databases, such as Scopus, Web of Science, Wiley Online Library, and Science Direct, as well as Google Scholar key words, such as ethnobotany and wild food plants, and words associated with each of the countries bordering the Mediterranean basin (Figure 3). The following key words and connectors were used: “Country” AND “ethnobotany” OR “ethnobotanical”, OR “food plants” OR “edible wild plants”. The publications were filtered for the English, Italian, and French languages, duplicates, document type (only peer-reviewed articles), and full text availability, and no chronological limits were applied in our search strategy. A simple evaluation of both title and abstract was carried out for every result in relation to the use of edible plants in human nutrition. The articles filtered in the previous point had their abstracts fully read in order to further reveal the real interest of the review article and to filter out non-applicable studies. The results thus obtained had their full contents read and evaluated. Only articles containing specific references to the use of edible flowers were included. Finally, an extensive evaluation of every document present in the “References” of the selected papers allowed us to gather further articles concerning ethnobotany or the wild plants used as food. As underlined by the authors of several scientific reviews, the criteria for article selection were devised a priori to avoid personal bias (e.g., [183,184]). In all, 380 articles were found in the databases as well as the previously collected papers, 112 of which contained reports of wild or cultivated edible flower uses. No data about the consumption of edible flowers were available for France, Montenegro, Syria, and Egypt. The nomenclature follows the World Flora Online [185]. We used the same electronic databases (included PubMed) to survey the phytochemical and clinical studies. The families are organized according to APG IV for angiosperms [186]. The abbreviations of the authors are standardized according to Brummitt and Powell [187], as recommended by Rivera et al. [188]. Based on the results obtained, we set up a database reporting the following data: taxon (when helpful, due to the recent changes in nomenclature, synonyms are reported in square parentheses), family, alimentary uses, country, and references. Vernacular names are provided in Table S1, Supplementary Materials.

## 5. Conclusions

The studies we included in this review demonstrate the established tradition in some countries of the Mediterranean basin of using wildflowers as part of the traditional diet. The phytochemical components of the edible flowers exhibit biological activities that can have a positive influence on health. The role of ethnobotanical studies is to avoid the loss of traditional knowledge concerning the use of food plants and, at the same time, to provide the basis for the development of new drugs from phytochemical and biochemical research.

In this regard, new field investigations aimed at the specific knowledge of edible flowers are desirable in the Mediterranean basin. Edible flowers may also have a great potential to become an important resource for profitable, integrated, local, and small-scale activities.

## Figures and Tables

**Table 1:** Traditionally edible flowers used in the Mediterranean basin. (Al = Albania; Bo-He = Bosnia-Herzegovina; Cr = Croatia; Gr = Greek; Is = Israel; It = Italy; Le = Lebanon; Mo = Morocco; Pa = Palestine; Sp = Spain; Tn = Tunisia; Tu = Turkey).

*Species* [Synonym]	Family	Uses	Country	References
*Acanthus hirsutus* Boiss.	Acanthaceae	Nectar as sweet	Tu	[23]
*Achillea collina* (Becker ex Rchb.f.) Heimerl	Asteraceae	Fritters	It	[24]
*Achillea millefolium* L.	Asteraceae	Liqueur. As vegetable	Bo-He, It	[25,26,27,28]
*Achillea moschata* Wulfen	Asteraceae	As flavoring, liqueur	It	[26,29]
*Achillea nana* L.	Asteraceae	Liqueur	It	[26]
*Acinos alpinus* (L.) Moench	Lamiaceae	Tea	Sp	[20]
*Alcea rosea* L.	Malvaceae	Juice	Ly	[30]
*Alliaria petiolata* (M.Bieb.) Cavara et Grande	Brassicaceae	As vegetable	It	[27,31]
*Allium ampeloprasum* L.	Amaryllidaceae	Seasoning	Sp	[24,32]
*Allium baeticum* Boiss.	Amaryllidaceae	Condiment	Tn	[33]
*Allium neapolitanum* Cirillo	Amaryllidaceae	Seasoning	It	[25]
*Allium roseum* L.	Amaryllidaceae	Condiment	Tn	[33]
*Anagyris foetida* L.	Fabaceae	Snack	It	[34]
*Anchusa azurea* Miller	Boraginaceae	Snack	Sp, Tu	[35,36]
*Anchusa undulata* L. subsp. * hybrida* (Ten.) Coutinho	Boraginaceae	Snack	Tu	[37]
*Anchusa italica* Retz.	Boraginaceae	Snack	Al	[38]
*Anchusa strigosa* Banks & Sol.	Boraginaceae	Snack	Tu	[39]
*Anethum graveolens* L.	Apiaceae	Liqueur	It	[40]
*Antennaria dioica* (L.) Gaertn.	Asteraceae	Tea	It	[41]
*Anthriscus nemorosa* (M.Bieb.) Sprengel	Apiaceae	As vegetable	Tu	[42]
*Aphyllanthes monspeliensis* L.	Liliaceae	NR	Sp	[43]
*Aquilegia vulgaris* L.	Ranunculaceae	Snack	Sp	[44]
*Arctium lappa* L.	Asteraceae	Jam	It	[26]
*Artemisia absinthium* L.	Asteraceae	Tea, liqueur	Bo-He, Sp	[20,28]
*Artemisia alba* Turra	Asteraceae	Seasoning	It	[34]
*Artemisia genipi* Stechm.	Asteraceae	Liqueur	It	[35,45,46,47,48]
*Artemisia glacialis* L.	Asteraceae	Liqueur	It	[31,45,46,47]
*Artemisia umbelliformis* subsp. *eriantha* (Ten.) Vallès-Xirau & Oliva Brañas	Asteraceae	Liqueur, as flavoring	It	[49]
*Artemisia vulgaris* L.	Asteraceae	Seasoning	It	[27]
*Asparagus stipularis* Forssk.	Asparagaceae	As vegetable	It	[50]
*Asphodelus albus* Mill. subsp. *subalpinus* Nyman	Asphodelaceae	Fritters, condiment	It	[50,51,52,53]
*Asphodelus ramosus* L. subsp. *ramosus*	Asphodelaceae	Fritters, condiment	It	[54,55,56,57,58]
*Bellardia trixago* (L.) All.	Plantaginaceae	Snack	Sp	[20]
*Bellis* spp. (*B. annua* L.; *B. perennis* L.)	Asteraceae	Tea. As vegetable	Bo-He, It, Tu	[27,28,59,60]
*Berberis vulgaris* L.	Berberidaceae	Snack	It	[26]
*Betula pendula* Roth.	Betulaceae	Mush, bread	Bo-He	[61]
*Bidens aurea* Sherff	Asteraceae	Tea	Sp	[62]
*Borago officinalis* L.	Boraginaceae	Snack, salads, vinegar aromatizer, fritters, soups	It, Ly, Sp	[24,27,30,39,42,53,63,64,65,66,67]
*Brassica fruticulosa* Cirillo	Brassicaceae	As vegetable	It	[59]
*Brassica incana* Ten.	Brassicaceae	As vegetable	It	[59]
*Brassica rupestris* Raf. subsp. *rupestris*	Brassicaceae	As vegetable	It	[59]
*Calendula officinalis* L., *C. arvensis* L.	Asteraceae	Aromatizer, salads	Bo-He, It	[24,26,27,28,33,48,68,69]
*Calligonum comosum* L’Her.	Polygonaceae	Eaten raw	Ly	[30]
*Calluna vulgaris* (L.) Hull.	Ericaceae	Spice	Bo-He	[61]
*Caltha palustris* L.	Ranunculaceae	As vegetable	Bo-He	[61]
*Capparis orientalis* Veill, *C. spinosa* L. [incl. C. ovata Desf.]	Capparaceae	Pickled, spice, condiment	Bo-He, Cr, Gr, It, Le, Tu	[27,33,61,64,66,70,71,72,73,74,75]
*Capsella bursa-pastoris* (L.) Medik	Brassicaceae	Eaten raw	Sp	[20]
*Carduus argyroa* Viv.	Asteraceae	As vegetable	It	[33]
*Carduus corymbosus* Ten.	Asteraceae	As vegetable	It	[33]
*Carduus nutans* L. subsp. *nutans*	Asteraceae	As vegetable	It	[27]
*Carlina acanthifolia* All.	Asteraceae	As vegetable	Al, It	[27,45,48,53,76,77]
*Carlina acaulis* L.	Asteraceae	As vegetable	It	[27,44,46,48,78,79,80]
*Carlina corymbosa* L.	Asteraceae	As vegetable	It	[33]
*Carlina gummifera* (L.) Less. [Atractylis gummifera L.]	Asteraceae	As vegetable	It	[55,56,57,58,81]
*Castanea sativa* Miller	Fagaceae	Liqueur	It	[40]
*Ceratonia siliqua* L.	Fabaceae	Fritters	It	[24]
*Cercis siliquastrum* L. subsp. *siliquastrum*	Fabaceae	Snack, fritters, condiment	It, Gr, Tu	[24,49,52,72,75,82,83]
*Cerinthe major* L.	Boraginaceae	Salads	It	[66,74]
*Chiliadenus glutinosus* Fourr. [Jasonia glutinosa (L.) DC.]	Asteraceae	Tea, liqueur	Sp	[20]
*Cirsium acaule* Scop.	Asteraceae	Snack	It	[46]
*Cirsium spinosissimum* (L.) Scop.	Asteraceae	Snack	It	[26,46]
*Convolvulus arvensis* L.	Convolvulaceae	Snack	It, Sp	[20,24]
*Corylus avellana* L.	Betulaceae	Mush, bread	Bo-He	[61]
*Corylus colurna* L.	Betulaceae	Mush, bread	Bo-He	[61]
*Cota altissima* (L.) J.Gay [Anthemis altissima L., A. arvensis L.]	Asteraceae	Tea	Sp, Tu	[35,84]
*Cota tinctoria* (L.) J.Gay [Anthemis tinctoria L.]	Asteraceae	Tea	Tu	[72]
*Cota wiedemanniana* (Fisch. & C.A.Mey.) Holub [Anthemis w. Fisch. & C.A.Mey.]	Asteraceae	Tea	Tu	[59,85]
*Crataegus monogyna* Jacq. subsp *monogyna*	Rosaceae	Liqueur, condiment	It	[26,67]
*Crataegus orientalis M.Bieb.* subsp. *orientalis*	Rosaceae	Eaten raw	Tu	[41,59,60]
*Crocus biflorus* Miller	Iridaceae	Seasoning	It	[33]
*Crocus longiflorus* Rafin.	Iridaceae	Seasoning	It	[57]
*Crocus neapolitanus* (Ker Gawl.) Loisel.	Iridaceae	Snack	It	[27]
*Crocus serotinus* Salisb.	Iridaceae	Condiment	Sp	[20]
*Crocus vernus* (L.) Hill.	Iridaceae	Snack, omelettes	It	[26,27]
*Cucurbita ficifolia* Bouche.	Cucurbitaceae	Fritters	Sp	[32]
*Cucurbita pepo* L. [incl.var. *oblonga*]	Cucurbitaceae	Sarma meal, meatball, fritters, sweet	Sp, Tu	[43,86]
*Cynara cardunculus* L. subsp. *cardunculus*	Asteraceae	Snack, fried	It	[58,78]
*Cynara horrida* Aiton	Asteraceae	As vegetable	It	[49]
*Cytinus hypocistis* (L.) L.	Rafflesiaceae	Snack	Sp	[20]
*Cynara humilis* L.	Asteraceae	As vegetable	Mo	[87]
*Dianthus seguieri* Vill. subsp. *requienii* (Godr.)	Caryophyllaceae	Liqueur	Sp	[42]
*Digitalis purpurea* L.	Plantaginaceae	Snack	It	[88]
*Digitalis thapsi* L.	Plantaginaceae	Snack	Sp	[20]
*Diplotaxis catholica* (L.) DC.	Brassicaceae	Eaten raw	Sp	[20]
*Dryas octopetala* L.	Rosaceae	Snack	It	[47]
*Echinophora tenuifolia* L.	Apiaceae	Soup, seasoning, beverages	Tu	[72]
*Echium creticum* L.	Boraginaceae	Snack	Sp	[20]
*Echium italicum* L.	Boraginaceae	Nectar as snack	Tu	[38]
*Echium plantagineum* L.	Boraginaceae	Nectar as snack	It, Sp	[20,67]
*Echium vulgare* L.	Boraginaceae	Snack	Sp	[20]
*Elaeagnus angustifolia* L.	Elaeagnaceae	Tea	Tu	[38]
*Ferula communis* L.	Apiaceae	As vegetable	Pa	[89]
*Foeniculum vulgare* Miller subsp. *vulgare*	Apiaceae	Seasoning	It	[24]
*Fragaria vesca* L.	Rosaceae	Liqueur	It	[39]
*Fritillaria lusitanica* Wikstr.	Liliaceae	Snack	Sp	[20]
*Fritillaria pyrenaica* L.	Liliaceae	Snack	Sp	[90]
*Fumaria capreolata* L. subsp. *capreolata*	Papaveraceae	As vegetable, snack	It	[45]
*Genista tridentata* L. [Pterospartum tridentatum (L.) Willk.]	Fabaceae	Tea	Sp	[20]
*Gentiana acaulis* L.	Gentianaceae	Liqueur	It	[25,47]
*Gentiana verna* L.	Gentianaceae	Liqueur	It	[25]
*Gladiolus byzantinus* Mill.	Iridaceae	Snack	It	[27,49]
*Gladiolus italicus* Miller	Iridaceae	Snack	It	[27]
*Gundelia tournefortii* L.	Asteraceae	As vegetable	Is, Pa	[89,91]
*Hedysarum coronarium* L.	Fabaceae	Salads	It	[24]
*Helichrysum italicum* (Roth) G. Don	Asteraceae	Tea, seasoning	Bo-He, Sp	[20,28]
*Helichrysum stoechas* (L.) Moench	Asteraceae	Tea, liqueur	Sp	[20]
*Hermodactylus tuberosus* (L.) Mill.	Iridaceae	Snack	It	[56,81]
*Herniaria glabra* L.	Caryophyllaceae	Tea, liqueur	Sp	[20]
*Hibiscus trionum* L.	Malvaceae	Spice, tea	Tu	[59,60]
*Hirschfeldia incana* (L.) Lagr.-Foss.	Brassicaceae	As vegetable	It	[58]
*Humulus lupulus* L.	Cannabinaceae	Spirits	Sp	[42]
*Hypericum perforatum* L.	Hypericaceae	Liqueur	Bo-He, It, Sp	[24,28,42]
*Iris persica* L.	Iridaceae	Snack	Tu	[38]
*Iris reticulata* M. Bieb	Iridaceae	Snack	Tu	[38]
*Iris sari* Schott ex Baker	Iridaceae	Eaten raw	Tu	[59]
*Isatis tinctoria* L. [incl. subsp. *canescens* (DC.) Arcang.]	Brassicaceae	As vegetable	It	[33,58]
*Jasonia tuberosa* (L.) DC.	Asteraceae	Tea	Sp	[20]
*Lamium album* L.	Lamiaceae	Snack, cakes	It	[26,79]
*Lamium bifidum* Cirillo	Lamiaceae	As vegetable	It	[27]
*Lamium galeobdolon* (L.) L.	Lamiaceae	Snack	Sp	[43]
*Lamium garganicum* L. subsp. *laevigatum* Arcang.	Lamiaceae	As vegetable	It	[27]
*Lamium maculatum* L.	Lamiaceae	As vegetable, sweet	It, Sp	[27,43,90]
*Lamium orvala* L.	Lamiaceae	Nectar as snack	It	[92]
*Lamium purpureum* L.	Lamiaceae	As vegetable	It, Tu	[23,27,93]
*Lantana camara* L.	Verbenaceae	Salads	Sp	[42]
*Larix decidua* Miller	Pinaceae	Liqueur	It	[26]
*Lathyrus sylvestris* L.	Fabaceae	As vegetable	It	[58]
*Lavandula angustifolia* L.	Lamiaceae	Seasoning, tea	It, Sp	[20,29,45]
*Lavandula latifolia* Medik	Lamiaceae	Seasoning, tea	Sp	[20]
*Lavandula pedunculata* L.	Lamiaceae	Seasoning, tea	Sp	[20]
*Lavandula stoechas* L.	Lamiaceae	Tea, liqueur	It, Sp	[20,49,57,94]
*Leontopodium nivale* (Ten.) Huet ex Hand.-Mazz	Asteraceae	Liqueur	It	[47]
*Leuzea conifera* DC.	Asteraceae	Tea. Eaten raw	Sp	[20]
*Linaria hirta* (L.) Moench	Plantaginaceae	Eaten raw	Sp	[20]
*Lithodora fruticosa* (L.) Griseb.	Boraginaceae	Snack	Sp	[20]
*Lonicera caerulea* L.	Caprifoliaceae	Snack	It	[27,47]
*Lonicera caprifolium* L.	Caprifoliaceae	Snack	It, Sp	[20,27]
*Lonicera implexa* Aiton	Caprifoliaceae	Snack	Sp	[20]
*Lonicera periclymenum* L.	Caprifoliaceae	Snack	Sp	[43]
*Malva neglecta* Wallr.	Malvaceae	Soup	It	[44]
*Malva sylvestris* L.	Malvaceae	Tea	Bo-He, Sp	[20,28]
*Matricaria aurea* (Loefl.) Sch.Bip. [Chamomilla aurea (Loefl.) Gay ex Cossom & Kralik]	Asteraceae	Tea	Ly, Pa	[30,89]
*Matricaria chamomilla* L. [Chamomilla recutita (L.) Rauschert]	Asteraceae	Tea, liqueur	Cr, It, Tu	[24,70,72,94,95,96]
*Melissa officinalis* L.	Lamiaceae	Tea. Spice, salads	It, Tu	[24,84]
*Mentha aquatica* L.	Lamiaceae	Spice	Tu	[84]
*Mentha gattefossei* Maire	Lamiaceae	Tea	Mo	[87]
*Micromeria juliana* (L.) Benth.	Lamiaceae	Seasoning	Tu, It	[57,86]
*Moltkia coerulea* Lehm.	Boraginaceae	As a sweet	Tu	[23]
*Narcissus poëticus* L.	Amaryllidaceae	As a sweet	It, Le	[46,97]
*Narcissus tazetta* L. subsp. *tazetta*	Amaryllidaceae	As vegetable	It	[56,57,58]
*Nigella arvensis* L. subsp. *glauca* (Boiss.) N.Terracc.	Ranunculaceae	Tea	Tu	[86]
*Nigritella rhellicani* Teppner & E. Klein [N. nigra (L.) Rchb.]	Orchidaceae	Seasoning	It	[26,45]
*Onobrychis humilis* (Loefl.) G.López	Fabaceae	Snack	Sp	[20]
*Ononis viscosa* L.	Fabaceae	Tea	Sp	[20]
*Onosma alborosea* Fisch. & C.A.Mey.	Boraginaceae	Nectar as snack	Tu	[38]
*Onosma roussaei* DC.	Boraginaceae	Nectar as snack	Tu	[38]
*Opuntia ficus indica* (L.) Miller [O. maxima Miller]	Cactaceae	Snack	Sp	[20]
*Papaver rhoaes* L.	Papaveraceae	Sorbet, patty, as a stew or egg–vegetable dish	Ly, Sp, Tu	[20,30,59,60,86]
*Pedicularis foliosa* L.	Orobanchaceae	Snack	It	[47]
*Pedicularis schizocalyx* (Lange) Steininger	Plantaginaceae	Snack	Sp	[90]
*Pentanema salicinum* (L.) D.Gut.Larr., Santos-Vicente, Anderb., E.Rico & M.M.Mart.Ort. [Inula salicina L.]	Asteraceae	Tea	Sp	[20]
*Periploca laevigata* Aiton	Asclepiadaceae	Snack	Sp	[20]
*Phlomis fruticosa* L.	Lamiaceae	Nectar as children’s snack	Cr	[70]
*Phlomis purpurea* L.	Lamiaceae	Snack	Sp	[20]
*Phlomis russeliana* (Sims) Lag. ex Benth.	Lamiaceae	Tea. Spice	Tu	[84]
*Pinus pinaster* Aiton	Pinaceae	Male flowers eaten raw	Sp	[20]
*Primula acaulis* (L.) Hill	Primulaceae	Salad	It, Sp	[29,90,92]
*Primula elatior* (L.) L.	Primulaceae	Snack	Sp	[90]
*Primula veris* L.	Primulaceae	Salads, snack, sweets	It, Sp	[25,26,27,32,39]
*Primula vulgaris* Hudson	Primulaceae	Snack	It	[27,53]
*Prunella grandiflora* (L.) Scholler	Lamiaceae	Snack	Sp	[20]
*Raphanus raphanistrum* L.	Brassicaceae	Eaten raw	Sp	[20]
*Robinia pseudoacacia* L.	Fabaceae	Snack, omelettes, fritters Syrup, liqueur	Bo-He, Cr, It, Sp	[24,26,28,29,32,39,44,45,47,48,53,61,64,65,77,79,83,94,98,99]
*Rosa* × *centifolia* L. [R. gallica var. centifolia (L.) Regel]	Rosaceae	Liqueur	Cr	[70]
*Rosa canina* L.	Rosaceae	Tea. Jam, syrup, snack, liqueur	It, Sp, Tu	[20,26,33,59,60]
*Rosa foetida* J. Herrm.	Rosaceae	Snack	Tu	[38]
*Rosa pouzinii* Tratt.	Rosaceae	Eaten raw	Sp	[20]
*Rubus ulmifolius Schott*	Rosaceae	Eaten raw	Sp	[20]
*Rumex roseus* L.	Polygonaceae	Salads	Tn	[63]
*Ruta graveolens* L.	Rutaceae	Soup	It	[39]
*Salvia officinalis* L.	Lamiaceae	Tea	Bo-He, Tu	[28,59]
*Salvia officinalis* L. subsp. *lavandulifolia* (Vahl) Gam [S. lavandulifolia Vahl]	Lamiaceae	Snack Liqueur	Sp	[20]
*Salvia rosmarinus* Schleid. [Rosmarinus officinalis L.]	Lamiaceae	Spice	Ly	[30]
*Salvia sclarea* L.	Lamiaceae	Jam	Tu	[23]
*Salvia tomentosa* Miller	Lamiaceae	Tea	Tu	[84]
*Salvia triloba* L. fil.	Lamiaceae	As vegetable	It	[49]
*Sambucus nigra* L.	Caprifoliaceae	Fried as a sweet, omelette, pancake, juice, seasoning, jam, jellies, beverages, vinegar aromatizer	Bo-He, Cr, It, Sp	[24,26,28,29,32,45,46,48,49,53,62,64,65,80,83,84,88,94,98]
*Sambucus racemosa* L.	Adoxaceae	Jams, jellies, fritters	It	[20,26,44,48]
*Santolina chamaecyparissus* L. s.l.	Asteraceae	Tea	Sp	[20]
*Santolina oblongifolia* Boiss.	Asteraceae	Tea	Sp	[20]
*Santolina rosmarinifolia* L.	Asteraceae	Tea	Sp	[20]
*Satureja montana* L.	Lamiaceae	Seasoning	Al, Bo-He	[28,77]
*Scolymus hispanicus* L.	Asteraceae	Seasoning	Sp	[35]
*Scorzonera undulata* Vahl subsp. *undulata*	Asteraceae	Salads	Tn	[63,100,101]
*Scrophularia trifoliata* L.	Plantaginaceae	Snack	It	[88]
*Sideritis hyssopifolia* L.	Lamiaceae	Tea	Sp	[62]
*Sideritis raeseri* Boiss. & Heldr.	Lamiaceae	Tea	Al	[102]
*Sideritis scardica* Griseb.	Lamiaceae	Tea	Al, It	[103,104]
*Silybum marianum* (L.) Gaertn	Asteraceae	Soup	Al, It	[103]
*Sinapis alba* L. subsp. *alba*	Brassicaceae	As vegetable	It	[59]
*Sinapis alba* L. subsp. *dissecta* (Lag.) Bonnier	Brassicaceae	As vegetable	It	[59]
*Sinapis arvensis* L.	Brassicaceae	As vegetable	It	[59]
*Sisymbrium officinale* (L.) Scop.	Brassicaceae	As vegetable	It	[59]
*Spartium junceum* L.	Fabaceae	Liqueur	Sp	[20]
*Stachys lavandulifolia* Vahl.	Lamiaceae	Tea	Tu	[60]
*Syzygium aromaticum* (L.) Merr. et Perry	Myrtaceae	Seasoning	Sp	[43]
*Tanacetum parthenium* (L.) Sch. Bip.	Asteraceae	To flavor vinegar	It	[24]
*Tanacetum vulgare* L.	Asteraceae	Tea	Sp	[20]
*Taraxacum campylodes* G.E.Haglund	Asteraceae	Jelly	It	[39]
*Taraxacum officinale* Weber & F.H. Wigg.	Asteraceae	Salads, fritters, jam, seasoning. Tea	Al, Cr, It, Tu	[26,29,47,66,72,79,94,105]
*Teucrium chamaedrys* L. subsp. *sinuatum* (Celak.) Rech. f.	Lamiaceae	Tea	Bo-He, Tu	[28,60]
*Teucrium montanum* L.	Lamiaceae	Tea	Bo-He	[28]
*Teucrium polium* L.	Lamiaceae	Spice	Tu	[59,60]
*Thymbra capitata* (L.) Cav. [Th. capitatus (L.) Hoffm.]	Lamiaceae	Seasoning	It, Ly	[30,33]
*Thymus atlanticus* (Ball) Roussine	Lamiaceae	Herbal drink	Mo	[87]
*Thymus hesperidum* Maire	Lamiaceae	Herbal drink	Mo	[87]
*Thymus saturejoides* Coss. & Balansa	Lamiaceae	Condiment	Mo	[87]
*Thymus vulgaris* L.	Lamiaceae	Herbal drink	Mo	[87]
*Thymus zygioides* Griseb	Lamiaceae	As spice	Tu	[84]
*Tilia cordata* Mill.	Malvaceae	Tea, liqueur	Bo-He, Cr	[20,62,93,95]
*Tilia platyphyllos* Scop.	Malvaceae	Tea, liqueur	Bo-He, Cr, Sp, Tu	[20,61,71,90,94]
*Tilia tomentosa* Moench [Tilia argentea Desf. ex DC.]	Malvaceae	Tea	Cr, Tu	[84,94]
*Tragopogon pratensis* L.	Asteraceae	Salads	It	[47,79,81]
*Tragopogon pterocarpus* DC.	Asteraceae	Eaten raw	Tu	[59]
*Trifolium alpinum* L.	Fabaceae	As vegetable	It	[25]
*Trifolium canescens* Willd.	Fabaceae	Jam	Tu	[106]
*Trifolium medium* L.	Fabaceae	Snack, cakes. Tea	It	[26]
*Trifolium pratense* L.	Fabaceae	Tea. Salads, cakes, fritters, soups	It, Sp, Tu	[20,24,25,26,35,45,79,90,106,107]
*Trifolium repens* L.	Fabaceae	Snack, flavoring	It	[24,26]
*Tripleurospermum parviflorum* (Wiild.) Pobed	Asteraceae	As a sweet	Tu	[23]
*Tropaeolum majus* L.	Tropaeolaceae	Preserved in vinegar, used as spice/vegetable	Cr	[70]
*Vachellia farnesiana* (L.) Wight & Arn [ Acacia f. (L.) Wild.]	Fabaceae	As vegetable	Ly	[30]
*Veronica allionii* Vill.	Plantaginaceae	Tea	It	[47]
*Veronica officinalis* L.	Plantaginaceae	Tea	It	[47]
*Vicia villosa* Roth	Fabaceae	Snack (nectar)	It	[67]
*Viola alba* Besser s.l.	Violaceae	Jam	Bo-He, It	[24,49,62,67,108]
*Viola alba* Besser subsp. *dehnhardtii* (Ten.) W.Becker	Violaceae	Salads, snack	It	[28,49]
*Viola biflora* L.	Violaceae	Seasoning	Bo-He,	[62]
*Viola canina* L. subsp. *canina*	Violaceae	As vegetable, sweets	It	[27,107]
*Viola bertolonii* Pio	Violaceae	As vegetable, sweets	It	[108]
*Viola etrusca* Erben	Violaceae	Salads	It	[27]
*Viola hirta* L.	Violaceae	Salads, snack, sweets	It	[27]
*Viola elegantula* Schott	Violaceae	Seasoning	Bo-He,	[62]
*Viola kitaibeliana* Roem. & Schult.	Violaceae	Snack Preserves	Gr	[76]
*Viola odorata* L.	Violaceae	Salads, sweet, fritters, liqueur	Bo-He, Cr, It, Sp	[20,25,28,39,45,47,61,99]
*Viola reichenbachiana* Jordan ex Boreau	Violaceae	Snack, preserves, candied fruit	Gr, It	[75,77,108]
*Viola tricolor* L.	Violaceae	Salads	Bo-He, It	[29,47,61,79]
*Zygophyllum fabago* L.	Zygophyllaceae	Brine (such as capers)	It	[52]
*Zygophyllum gaetulum* Emb. & Maire	Zygophyllaceae	Tea	Mo	[87]
*Zygophyllum waterlotii* Maire	Zygophyllaceae	Tea	Mo	[87]

## Data Availability

All the relevant data used for the paper can be found in Table 1.

## Supplementary Materials

The following is available online at https://www.mdpi.com/article/10.3390/plants11233272/s1, Table S1: Vernacular names of the traditionally edible flowers used in the Mediterranean basin.
