# Supplementary material for: Edible Flowers Used in Some Countries of the Mediterranean Basin: An Ethnobotanical Overview
Source: Plants (Basel). 2022 Nov 28;11(23):3272. doi: 10.3390/plants11233272 (PMC9736219; doi:10.3390/plants11233272)
Supplement: Supplementary file 1 [file plants-11-03272-s001.zip › plants-2022619-supplementary.pdf]

Table S1 Vernacular names of the traditionally edible flowers used in the Mediterranean basin. (Al= Albany; Bo-He= Bos-nia-Herzegovina; Cr= Croatia; Gr.= Greek; Is=Israel; It= Italy; Le=Lebanon; Mo=Morocco; Pa= Palestine; Sp= Spain; Tn= Tunisia; Tu= Turkey.)

**Species**	**Vernacular names**
Acanthus hirsutus Boiss.	Ökütz osurtan (Tu)
Achillea collina (Becker ex Rchb.f.) Heimerl	Millefoje, stagnasangue (It)
Achillea millefolium L.	Hajdučka trava, Stolisnjak, Sporiš (Bo-He); Erba besa, Millefoglie, Moufètte, Fleur blanc, Tanéda mata, Tanédón, Livia mata, Milafior,Lívia di préi, Carèdiverm, Danéda mata (It)
Achillea moschata Wulfen	Livia, Tanéda, Danéda (It)
Achillea nana L.	Tanéda, Tanéda mata, Tanéda bas, Livia di ges (It)
Acinos alpinus (L.) Moench	Té de sierra, Té del monte (Sp)
Alcea rosea L.	N.R.
Alliaria petiolata (M.Bieb.) Cavara et Grande	N.R.
Allium ampeloprasum L.	Ajete, Ajo silvestre, Puerro, Puerro silvestre (Sp)
Allium baeticum Boiss.	Korath (Tn)
Allium neapolitanum Cirillo	Yazoul, Gazoul, Azoul (Tn)
Allium roseum	Korath (Tn)
Anagyris foetida L.	Fasolu taddi (It)
Anchusa azurea Miller	Chupamiel, Alcalcuz (Sp); Mijmejok (Tu)
Anchusa undulata L. subsp. *hybrida* (Ten.) Coutinho)	N.R.
Anchusa italica Retz.	Alkanet (Al)
Anchusa strigosa Banks & Sol.	Hımhım, Gûrîz (Tu)
Anethum graveolens L.	Aneto (It)
Antennaria dioica (L.) Gaertn.	N.R.
Anthriscus nemorosa (M.Bieb.) Sprengel	Xitok (Tu)
Aphyllanthes monspeliensis L.	Llonses (Sp)
Aquilegia vulgaris L.	Kukufraka (Sp)
Arctium lappa L.	Nápula, Nápol, Moros,Berdèna, Grignapola (It)
Artemisia absinthium L.	Pelin (Bo-He); Axenxo, Donzell (Sp)
Artemisia alba Turra	Canforedda (It)
Artemisia genipi Stechm.	Genepì, Genepì giallo, Genepì maschi, Gënëpi fumél (It)
Artemisia glacialis L.	Genepì fumelo, Bava dei ghiacciai (It)
Artemisia umbelliformis subsp. *eriantha* (Ten.) Vallès-Xirau & Oliva Brañas	Genepì (It)
Artemisia vulgaris L.	N.R.
Asparagus stipularis Forssk.	N.R.
Asphodelus albus Mill. subsp. *subalpinus* Nyman	N.R.

Asphodelus ramosus L. subsp. *ramosus*	Asfodelo, Porraccio, Purrazzu, Arvùzzi ramùsi (It)
Bellardia trixago (L.) All.	Torta de pastor (Sp)
Bellis spp. *(B. annua* L.; *B. perennis* L.)	Ovčica (Bo-He); Papatya (Tu)
Berberis vulgaris L.	Špin d'uĝét, Spin d'asen, Feruda, Crispin (It)
Betula pendula Roth.	Breza (Bo-He)
Bidens aurea Sherff	Té, Té americano, Té chino, Té castellano, Té de Buenos Aires, Té de huerta, Té de huerto, Té de M´ejico, Té de Canarias, Té moro, Té moruno (Sp)
Borago officinalis L.	Borratja (Sp); Borracce, Borragia, Borragine, Borrascine, Burracce, Burraccedde, Burraccelle, Burracchia, Burraccia, Burrascene, Burrasciana, Burrascina, Ferrascene, Murraine, Pezze De Iarde, Sucamele, Suzzamele, Verrascene, Verrascene , Vorraina, Vorraine, Vurraina, Vurraine, Vurrascene (It)
Brassica fruticulosa Cirillo	Caulicèddu, Qualicèddu (It)
Brassica incana Ten.	Amarèddi (It)
Brassica rupestris Raf. subsp. *rupestris*	Cavulazzu, Càulu di rocca (It)
Calendula officinalis L., *C. arvensis* L.	Neven (Bo-He); Calendula, Calennula, Kalendula (It)
Calligonum comosum L'Her.	N.R.
Calluna vulgaris (L.) Hull.	Vris (Bo-He)
Caltha palustris L.	Kaljužnica (Bo-He)
Capparis orientalis Veill, *C. spinosa* L. [incl. C. ovata Desf.]	Kapar (Bo-He); Kapare (Cr); Cappero, Chiapparu, Chiapperi, Chiapparedda (It); Kabbar (Tn); Gebere, Dikenli gebere (Tu)
Capsella bursa-pastoris (L.) Medik	Devanaeras, Chorrontelas (Sp)
Carduus argyroa Viv.	Napodi d'acqua, Napordio (It)
Carduus corymbosus Ten.	Carvi (It)
Carduus nutans L. subsp. *nutans*	N.R.
Carlina acanthifolia All.	Cardéla, Carlina (It)
Carlina acaulis L.	Carlina, Garzun, Cardan, Cardòn, Cardon plàt, Chardousso, Spinus (It)
Carlina corymbosa L.	Mazzacani (It)
Carlina gummifera (L.) Less.	Masticògna, Masticògna, Cacucciulìdda (It)
Castanea sativa Miller	Castagna, Cjastinis, Kostànj (It)
Ceratonia siliqua L.	Carruba, Carrobie (It)
Cercis siliquastrum L. subsp. *siliquastrum*	Koutsipia, Redbud (Gr); Albero di Giuda (It)
Cerinthe major L.	Sucamele (It)
Chiliadenus glutinosus Fourr. [Jasonia glutinosa (L.) DC.]	Té de roca, Té de peña, Arnica (Sp)
Cirsium acaule Scop.	Vazanel (It)
Cirsium spinosissimum (L.) Scop.	Chardousso, Ĝardón, Atición dalción, Sc'pín, Cardón de lirozocola (It)
Convolvulus arvensis L.	Campanelle (It); Correhuela (Sp)
Corylus avellana L.	Lijeska (Bo-He)

Corylus colurna L.	Meija lijeska (Bo-He)
Cota altissima (L.) J.Gay [Anthemis altissima L., A. arvensis L.]	Magarza, gamarza (Sp); Büyük papatya (Tu)
Cota tinctoria (L.) J.Gay [Anthemis tinctoria L.]	N.R.
Cota wiedemanniana (Fisch. & C.A.Mey.) Holub [Anthemis w. Fisch. & C.A.Mey.]	Papatya (Tu)
Crataegus monogyna Jacq. subsp monogyna	Bianc de špín, Špín, Biancospín (It)
Crataegus orientalis M.Bieb. subsp. *orientalis*	Guvij, Heluje, Sez (Tu)
Crocus biflorus Miller	Ciuri pi fari u zafferanu (It)
Crocus longiflorus Rafin.	N.R.
Crocus neapolitanus (Ker Gawl.) Loisel.	N.R.
Crocus serotinus Salisb.	Azafrán (Sp)
Crocus vernus (L.) Hill.	Ćiĝámola, Cigámbola deldiául, Cigambula, Bucanef (It)
Cucurbita ficifolia Bouche.	Calabaza, Calabazo, Calabaza de cabello de ángel (Sp)
Cucurbita pepo L. [incl.var. *oblonga*]	Flor de carabassó (Sp); Kızartmalık kabak (Tu)
Cynara cardunculus L. subsp. *cardunculus*	Cardùn'i spini, Cacòcciuliddu spinusu (It)
Cynara horrida Aiton	N.R.
Cytinus hypocistis (L.) L.	Melera, Mamelletes (Sp)
Cynara humilis L.	Timet (Mo)
Dianthus seguieri Vill. subsp. *requienii* (Godr.)	Clavells de bosc (Sp)
Digitalis purpurea L.	Sa poddigale (It)
Digitalis thapsi L.	Chupador (Sp)
Diplotaxis catholica (L.) DC.	Pan y quesito amarillo (Sp)
Dryas octopetala L.	N.R.
Echinophora tenuifolia L.	N.R.
Echium creticum L.	Chupamieles (Sp)
Echium italicum L.	Hımhım, Mijok (Tu)
Echium plantagineum L.	Suzzamele (It); Argamula, Chupamieles (Sp)
Echium vulgare L.	Chupamiel, Bovina (Sp)
Elaeagnus angustifolia L.	Sinç (Tu)
Ferula communis L.	N.R.
Foeniculum vulgare Miller subsp. *vulgare*	Finocchio selvatico, finocchio cavallì, finocchietto (It)
Fragaria vesca L.	Fragolina, Jaguca (It)
Fritillaria lusitanica Wikstr.	Campanicas (Sp)
Fritillaria pyrenaica L.	Jarras (Sp)
Fumaria capreolata L. subsp. *capreolata*	Fumàa, Cantagalétti (It)

Genista tridentata L. [Pterospartum tridentatum (L.) Willk.]	Carquesa, Carquexa (Sp)
Gentiana acaulis L.	Genzianella, Gentièn, Peirette, Braio d'cucuc, Pirulet (It)
Gentiana verna L.	Genzianella, Gentièn, Fieur di Corbas (It)
Gladiolus byzantinus Mill.	N.R.
Gladiolus italicus Miller	N.R.
Gundelia tournefortii L.	A'kub (Is)
Hedysarum coronarium L.	Lupinella, Lupina (It)
Helichrysum italicum (Roth) G. Don	Cmilje, Smilje (Bo-He); Manzanilla silvestre (Sp)
Helichrysum stoechas (L.) Moench	Mançanilla borda, Manzanilla (Sp)
Hermodactylus tuberosus (L.) Mill.	N.R.
Herniaria glabra L.	Manzanilla del campo (Sp)
Hibiscus trionum L.	Hatmi cicegi (Tu)
Hirschfeldia incana (L.) Lagr.-Foss.	Làssimi, Mazzareddi (It)
Humulus lupulus L.	Llúpol (Sp)
Hypericum perforatum L.	Gospina trava (Bo-He); Scacciadiavoli, Erba di S. Giovanni (It); Herba de sant Joan (Sp)
Iris persica L.	Birbîzêk, Birbîzêka mîha, Birxızeylê (Tu)
Iris reticulata M. Bieb	Birbîzêk, Birbîzêka bizina, Birxızeylê (Tu)
Iris sari Schott ex Baker	Nergiz (Tu)
Isatis tinctoria L. [incl. subsp. *canescens* (DC.) Arcang.]	Cavolu carrammu, Guàdu (It)
Jasonia tuberosa (L.) DC.	Té, Té de tierra (Sp)
Lamium album L.	Ortíĝa máta, Ortía máta (It)
Lamium bifidum Cirillo	N.R.
Lamium galeobdolon (L.) L.	N.R.
Lamium garganicum L. subsp. *laevigatum* Arcang.	N.R.
Lamium maculatum L.	N.R.
Lamium orvala L.	N.R.
Lamium purpureum L.	Emzikotu (Tu)
Lantana camara L.	Caputxina (Sp)
Larix decidua Miller	Láreš, Láraš, Larĝá, Larĝé (It)
Lathyrus sylvestris L.	Cessavuoi, Fasòla sarvaggia (It)
Lavandula angustifolia L.	Steccadò, Cadò (It); Spígol (Sp)
Lavandula latifolia Medik	Alhucema, Espígol (Sp)
Lavandula pedunculata L.	Antueso (Sp)
Lavandula stoechas L.	Spicu, Ispigu (It); Cantueso, Cap díase, Bofarull (Sp)
Leontopodium nivale (Ten.) Huet ex Hand.-Mazz	Stela alpina (It)
Leuzea conifera DC.	Cardo santo, Alcachofilla (Sp)
Linaria hirta (L.) Moench	Pan y queso (Sp)
Lithodora fruticosa (L.) Griseb.	Sietesangrías (Sp)
Lonicera caerulea L.	N.R.

Lonicera caprifolium L.	N.R.
Lonicera implexa Aiton	Chupaores (Sp)
Lonicera periclymenum L.	Jesu kristoren atzamarra, Jesu kristoren eskue, Jaungoikoan eskue (Sp)
Malva neglecta Wallr.	Malva selvadiga (It)
Malva sylvestris L.	Crnisljez, Plavisljez, Vučja stopa (Bo-He); Malva (Sp)
Matricaria aurea (Loefl.) Sch.Bip. [Chamomilla aurea (Loefl.) Gay ex Cossom & Kralik]	Babounej (Ly)
Matricaria chamomilla L. [Chamomilla recutita (L.) Rauschert]	Kamomila, Kamilica (Bo-He); Capumilla (It)
Melissa officinalis L.	N.R.
Mentha aquatica L.	Ogul otu (Tu)
Mentha gattefossei Maire	Flyou dial jbel (Mo)
Micromeria juliana (L.) Benth.	Kekik (Tu)
Moltkia coerulea Lehm.	N.R.
Narcissus poëticus L.	Susarelo, Joes de mel, Fior del mel (It)
Narcissus tazetta L. subsp. *tazetta*	Narcìsu, Agghi porri (It)
Nigella arvensis L. subsp. *glauca* (Boiss.) N.Terracc.	Çay bitkisi, Verdasfar, Gulşîlave zer (Tu)
Nigritella rhellicani Teppner & E. Klein [N. nigra (L.) Rchb.]	Moréti, Morétino, Moréto, Man dela madona, Manina dela Madòna, Man del Signór, Vaniglione (It)
Onobrychis humilis (Loefl.) G.López	Sangre de dios (Sp)
Ononis viscosa L.	Gorromino (Sp)
Onosma alborosea Fisch. & C.A.Mey.	Hımhım, Mîjok (Tu)
Onosma roussaei DC.	Hımhım, Mîjok (Tu)
Opuntia ficus indica (L.) Miller [O. maxima Miller]	Chumbera, Palas (Sp)
Papaver rhoaes L.	Amapola, Babaol, Rosella, Ruelles (Sp); Bük, Gelincik, Gelincik, Lale, Ruja, Gül, Nöninka, Laninka (Tu)
Pedicularis foliosa L.	N.R.
Pedicularis schizocalyx (Lange) Steininger	N.R.
Pentanema salicinum (L.) D.Gut.Larr., Santos-Vicente, Anderb., E.Rico & M.M.Mart.Ort. [Inula salicina L.]	N.R.
Periploca laevigata Aiton	N.R.
Phlomis fruticosa L.	N.R.
Phlomis purpurea L.	Matagallo (Sp)
Phlomis russeliana (Sims) Lag. ex Benth.	Ari otu (Tu)
Pinus pinaster Aiton	Pino rodeno (Sp)
Primula acaulis (L.) Hill	Coucù, Fiou ed mortz, Pamparà, Primula (It)

Primula elatior (L.) L.	N.R.
Primula veris L.	Cuculucia, Primola, Galet, Fior de san giusef, Calet (It); Flor de mayo, Flor de SanJosé, Pámpano (Sp)
Primula vulgaris Hudson	Viole di pane, Fiori di pane, Erba di S. Antonio (It)
Prunella grandiflora (L.) Scholler	Herba melera (Sp)
Raphanus raphanistrum L.	Jaramago (Sp)
Robinia pseudoacacia L.	Bagrem (Bo-He); Kacia, Bagrem (Cr); Acacia, Acasia, Akàcja, Gazhillo (It); Azkazia, Acacia, Pan y quesito, Pan y queso (Sp)
Rosa × centifolia L. [R. gallica var. centifolia (L.) Regel]	Vonjača, čentifolia, Ruža (Bo-He)
Rosa canina L.	Špin de frósola, Róśa salvádia, Špin de anfrósula, Rosa selvadega, Frósola, Frósula (It); Tapaculos, Escaramujo (Sp); Sırgul, Silan (Tu)
Rosa foetida J. Herrm.	Verdasfar, Gulşîlave zer (It)
Rosa pouzinii Tratt.	Escaramujo (Sp)
Rubus ulmifolius Schott	Zarza, Zarzamora, Silva, Esbarzer (Sp)
Rumex roseus L.	Hommitha (Tn)
Ruta graveolens L.	Ruta, Grutca (It)
Salvia officinalis L.	Kadulja ,Pelin (Bo-He); Adaçavı (Tu)
Salvia officinalis L. subsp. *lavandulifolia* (Vahl) Gam [S. lavandulifolia Vahl]	Salvia, Savia (Sp)
Salvia rosmarinus Schleid. [Rosmarinus officinalis L.]	N.R.
Salvia sclarea L.	Ayı kulağı, Arıotu (Tu)
Salvia tomentosa Miller	Adaçaı (Tu)
Salvia triloba L. fil.	N.R.
Sambucus nigra L.	Bazga, Zoha, Zova (Bo-He); Armul, Sambuco, Bazovina, Paparozzo, Sambúch, Sambúch neir, Sambügu, Sèuc, Confecc, Savuco, Sauco, Pastaccera, Savuci, Savucio, Samuhu (It); Sabuco, Saúco (Sp)
Sambucus racemosa L.	Sambūch salvádik, Sambúch ros, Sambúch mat, Sèuc, Soré (It)
Santolina chamaecyparissus L. s.l.	Camamilla, Camamirla, Manzanilla, Salvaje (Sp)
Santolina oblongifolia Boiss.	Manzanilla de gredos (Sp)
Santolina rosmarinifolia L.	Manzanilla blanca (Sp)
Satureja montana L.	Vrijesak (Bo-He)
Scolymus hispanicus L.	Cardillo (Sp)
Scorzonera undulata Vahl subsp. *undulata*	Chtel, guiz (Tn)
Scrophularia trifoliata L.	N.R.
Sideritis hyssopifolia L.	Té, Té amarillo, Té borriquero, Té de bosc, Té de botja, Té d'espiga, Té de Galba, Té de glera, Té de la peña, Té de lastra, Té de matxo, te de muntanya, Té de monte, Té de pastor, Té de peña, Té de Picos de Europa, Té de Picos, Té de piedra, Té de puerto, Té de roca, t´e de tasca, Té fino, te negre, Té purgante, Té rastrero, Té salvatge, Té verdadero (Sp)
Sideritis raeseri Boiss. & Heldr.	Çaj, Çaj të bardhë, Çaj fushe (Al)
Sideritis scardica Griseb.	Çaj (Al)
Silybum marianum (L.) Gaertn	Cardone (It)

Sinapis alba L. subsp. *alba*	Làssani, Mazzarèddu (It)
Sinapis alba L. subsp. *dissecta* (Lag.) Bonnier	Sinacciòlu di linu (It)
Sinapis arvensis L.	Alàssani, Sinàpa sarvaggia (It)
Sisymbrium officinale (L.) Scop.	Làssinu di sceccu, Mazzarèddri (It)
Spartium junceum L.	Ginesta (Sp)
Stachys lavandulifolia Vahl.	Çaye qwe, Deme çole (Tu)
Syzygium aromaticum (L.) Merr. et Perry	Clau (Sp)
Tanacetum parthenium (L.) Sch. Bip.	Matrecara, erba amara (It)
Tanacetum vulgare L.	Tanarides (Sp)
Taraxacum campylodes G.E.Haglund	Talla, Talis, Lidrichessa, Modac (It)
Taraxacum officinale Weber & F.H. Wigg.	Divlia radice, maslacac (Cr);Atación, Insaláta di pra', Dent da prà, Dent de can, Amarogghie, Cicuriedde, Cicuriùne, Fogghia resta, Fogghja de fore, Piscialette, Tarassache, Zangone, Zangune restù (It)
Teucrium chamaedrys L. subsp. *sinuatum* (Celak.) Rech. f.	Dubačac (Bo-He)
Teucrium montanum L.	Ivatrava (Bo-He)
Teucrium polium L.	Ürper (Tu)
Thymbra capitata (L.) Cav. [Th. capitatus (L.) Hoffm.]	Sataredda (It)
Thymus atlanticus (Ball) Roussine	Azukenni (Mo)
Thymus hesperidum Maire	Azukenni (Mo)
Thymus saturejoides Coss. & Balansa	Azukenni (Mo)
Thymus vulgaris L.	Zaâtar (Mo)
Thymus zygioides Griseb	Dag kekik (Tu)
Tilia cordata Mill.	Sitnolisna lipa (Bo-He); Lìpa (Cr); Tiller (Sp)
Tilia platyphyllos Scop.	Krupnolisna lipa (Bo-He); Tilo, Tiller (Sp); Ihlamu (Tu)
Tilia tomentosa Moench [Tilia argentea Desf. ex DC.]	Lipa (Cr); Ihlamur (Tu)
Tragopogon pratensis L.	Barba di capra, Barbouch, Barbabouch (It)
Tragopogon pterocarpus DC.	Yemlik (Tu)
Trifolium alpinum L.	Trifoglio di montagna, Seutrin, Trioùla, Sanfuèn
Trifolium canescens Willd.	Yonca Otu, Çayırotu (Tu)
Trifolium medium L.	Trefól, Trifogl (It)
Trifolium pratense L.	Sciuada madonna, Suria Pane del latte (It); Chubador, Trébol (Sp); Yonca Otu, Çayırotu (Tu)
Trifolium repens L.	Trefól, Trefoil blanc (It)
Tripleurospermum parviflorum (Wiild.) Pobed	Papatya (Tu)
Tropaeolum majus L.	Ljubidrag, Dragušac (Bo-He)
Vachellia farnesiana (L.) Wight & Arn .[Acacia f. (L.) Wild.].	N.R.

Veronica allionii Vill.	Èrbë d'tè, Giaspertere, Té d'mountannho (It)
Veronica officinalis L.	Èrbë d'tè, Tè svizzero (It)
Vicia villosa Roth	Pane e casu (It)
Viola alba Besser s.l.	Bijela ljubiica, Bijela djetelina (Bo-He); Violetta (It)
Viola alba Besser subsp. *dehnhardtii* (Ten.) W.Becker	N.R.
Viola biflora L.	Dvocvjetna ljubi1ica (Bo-He)
*Viola canina*L. subsp. *canina*	N.R.
Viola bertolonii Pio	N.R.
Viola etrusca Erben	N.R.
Viola hirta L.	N.R.
Viola elegantula Schott	Lijepa ljubi1ica (Bo-He)
Viola kitaibeliana Roem. & Schult.	Violet (Gr)
Viola odorata L.	Ljubica mirisna (Bo-He); Viola, Violetta (It); Violeta (Sp)
Viola reichenbachiana Jordan ex Boreau	Violet (Gr)
Viola tricolor L.	Danino, maouhica (Bo-He); Violette, Viooulëtìn, Vioulëtto blancho (It)
Zygophyllum fabago L.	N.R.
Zygophyllum gaetulum Emb. & Maire	Lâagaya (Mo)
Zygophyllum waterlotii Maire	Lâagaya (Mo)
